# Comparing the accuracy of brief versus long depression screening instruments which have been validated in low and middle income countries: a systematic review

**DOI:** 10.1186/1471-244X-12-187

**Published:** 2012-11-01

**Authors:** Dickens Akena, John Joska, Ekwaro A Obuku, Taryn Amos, Seggane Musisi, Dan J Stein

**Affiliations:** 1 Department of Psychiatry and Mental Health, University of Cape Town, Cape Town, South Africa; 2 Department of Psychiatry, Makerere University, Kampala, Uganda; 3 Joint Clinical Research Centre, Kampala, Uganda

## Abstract

**Background:**

Given the high prevalence of depression in primary health care (PHC), the use of screening instruments has been recommended. Both brief and long depression screening instruments have been validated in low and middle income countries (LMIC), including within HIV care settings. However, it remains unknown whether the brief instruments validated in LMIC are as accurate as the long ones.

**Methods:**

We conducted a search of PUBMED, the COCHRANE library, AIDSLINE, and PSYCH-Info from their inception up to July 2011, for studies that validated depression screening instruments in LMIC. Data were extracted into tables and analyzed using RevMan 5.0 and STATA 11.2 for the presence of heterogeneity.

**Results:**

Nineteen studies met our inclusion criteria. The reported prevalence of depression in LMIC ranged from 11.1 to 53%. The area under curve (AUC) scores of the validated instruments ranged from 0.69-0.99. Brief as well as long screening instruments showed acceptable accuracy (AUC≥0.7). Five of the 19 instruments were validated within HIV settings. There was statistically significant heterogeneity between the studies, and hence a meta-analysis could not be conducted to completion. Heterogeneity chi-squared = 189.23 (d.f. = 18) p<.001.

**Conclusion:**

Brief depression screening instruments in both general and HIV-PHC are as accurate as the long ones. Brief scales may have an edge over the longer instruments since they can be administered in a much shorter time. However, because the ultra brief scales do not include the whole spectrum of depression symptoms including suicide, their use should be followed by a detailed diagnostic interview.

## Background

Depression is a prevalent and disabling condition in both high and low income countries [1-3]. According to the World Health Organization, depression is the 4^th^ most disabling medical disorder, and is predicted to be the 2^nd^ most disabling medical condition by 2020 [1,4]. The 12-month prevalence of depression has been reported as 4.1%, with a lifetime prevalence of 6.7% [5].

Treatment guidelines developed in high income countries (HIC) recommend routine screening for depression in primary health care (PHC) as an initial step in holistic patient care [6-8]. A number of brief (≤12 items) instruments including the patient health questionnaire (PHQ-9) [9,10] and the Kessler-10 (K-10) [11] have been validated in low and middle income countries (LMIC). Similarly, longer (≥15 items) instruments including the centre for epidemiological studies-depression (CES-D) [12] have also been validated in LMIC.

The bulk of research summarizing findings about the accuracy of validated depression screening instruments has come from HIC, providing conflicting data [13-15]. For example, one review found marginal differences between brief and ultra-brief scales [14], while a meta-analysis by Mitchell et al. (2007) reported that brief and ultra-brief scales were equally accurate [15].

Generalizing findings from studies conducted in HIC to LMIC may be inappropriate due to a number of differences. Low literacy rates, cultural diversity and high patient numbers are some factors that are unique to LMIC [3,16,17]. Such differences as low literacy rates may influence the accuracy of depression screening instruments, making the generalization of findings from HIC to LMIC the more difficult.

Depression is a major health problem across LMIC; however, a number of countries in sub-Saharan Africa are equally plagued with a high burden of HIV/AIDS. Indeed close to two thirds of all persons living with HIV/AIDS (PLWHA), reside in sub-Saharan Africa [18]. Research has also shown that up to 30% of PLWHA may develop depressive disorder during the course of their illness [19,20].

The screening of depression among PLWHA is important for a number of reasons; the presence of symptom overlap between the two disorders being one of them. For example, suicide, fatigue, sadness and insomnia are symptoms reported by both PLWHA and those with depression. The existence of symptom overlaps call for screening PLWHA who present at PHC for depression. Indeed a number of researchers have recommended the routine screening of depression in PLWHA [21-24]. However, literature about the validity of screening instruments in the setting of HIV/AIDS remains scanty [25].

The aim of our systematic review was to examine the accuracy of depression screening instruments which have been validated in LMIC, comparing brief and long scales. We also compared the accuracy of instruments validated in general and HIV-PHC settings.

These findings could guide clinicians about which scales to adapt for routine use in busy PHC settings within LMIC.

## Methods

A literature search was conducted using the following approach:

We searched the PUBMED, COCHRANE library, AIDSLINE, and PSYCH-Info databases for studies published in English from inception up to July 2011. In our search, we used the following key words: sensitivity/specificity, validation, depression/depressive disorders, and screening instruments/tools/scales. These key words were combined with LMIC, HIV/AIDS, Africa, Asia, Eastern Europe, and South America. We then searched reference lists from retrieved articles for suitable papers and consulted two sets of authors [26,27] for more clarity regarding data in their papers.

### Study selection

Studies were included if they had the following outcomes of interest:

1. A depression screening instrument followed by a formal diagnostic instrument or an interview was administered to all screened patients i.e. both screen positive and negatives.

The diagnosis of a depressive disorder(major/minor/dysthymia) was based on the ICD-10 [28], DSM-IV [29], or an instrument frequently used as a gold standard. Instruments routinely used to screen for depression including the [30,31] were not considered gold standard, even though a number of studies had used them [25,32].

2. Studies were conducted in non-mental health facilities

3. Studies reported the sensitivity, specificity, the AUC and predictive values of the screening instrument in comparison to the diagnostic standard.

4. Studies were conducted in LMIC as defined by the world bank [33].

### Data analysis

Data from included studies was extracted by one author (DA) into tables constructed in MS Excel, and later transferred to RevMan version 5.1.2 [34]. We used RevMan to construct a diagnostic 2x2 table by calculating the true positive, false positive, false negative and true negative figures from the sensitivity/specificity and prevalence values provided in all the included studies. The figures from the 2x2 tables generated using RevMan were then fitted in STATA version 11.2 [35] to assess for heterogeneity using random effects analysis model. Assessing for heterogeneity guided us, as to whether it was possible to pool, analyze, and report the findings as a meta-analysis. We used meta-analytic commands in STATA for the analysis.

### Study quality assessment and inclusion

**Figure 1:**
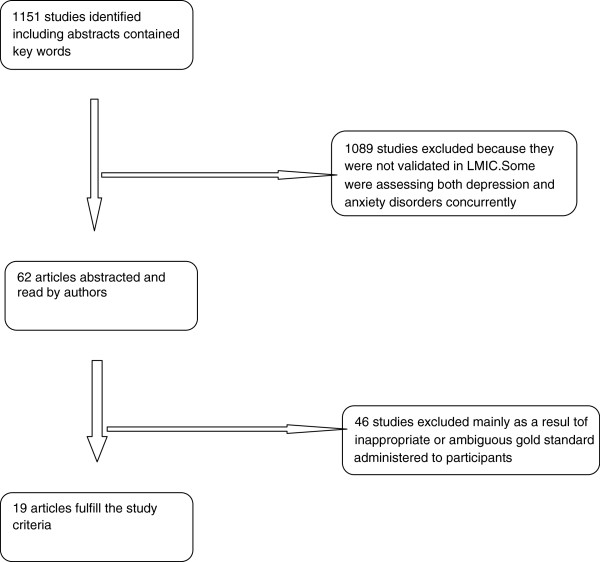
Study selection process for the systematic review.

Data was independently abstracted by three authors (DA, EO and TA). DA read all the abstracts, 1151 studies were excluded based on abstracts alone. Full articles for 65 articles were identified for further scrutiny. Of the 65 articles identified for further scrutiny, 14 studies in which 19 instruments were validated with 3759 participants met our criteria. See Figure 1.

Study inclusion and exclusion was independently done by DA, EO and TA, in the event of ambiguity, DJS was the arbitrator. We used RevMan to assess study quality. The parameters assessed included blinding of reference information from screening results, screening of patients from highly selected populations, and selection of who gets the gold standard from among a screened population. Study quality was rated as fair, acceptable and good quality. All included studies were then scrutinized independently by JJ.

## Results

Of the 19 included studies, 10 fulfilled all the reporting criteria by RevMan [30] and were considered of good quality [26,36-42].One study was considered fair in quality due to the lack of blinding and referral of only screen positives for the diagnosis from a highly selected population [11]. The rest of the studies (n=8) were considered acceptable. The studies with acceptable quality had limited information about blinding, some lacked clarity about the time interval between administration of the screening instrument and gold standard [27,43-47].

### General description of studies

Eleven studies were conducted in Africa [11,26,27,38,40-43,47], five of which were in HIV settings [26,27,38,41,43]. Two studies were conducted in South America [36,37] and six in Asia [39,44-46] The most frequently used diagnostic instrument was the mini international neuropsychiatric instrument (MINI) [48]. Table 1 below shows the general characteristics of the studies. The sample sizes of included studies ranged from 61 to 649. The prevalence of depression varied widely across populations ranging from 11.1 to 53.5% (see Table 2 below). There were also wide variations within continents, and also according to the different instruments used. All validated instruments were able to adequately identify depression, with AUC ranging from 0.69-0.99. Table 2 above shows the variables that were used to assess for heterogeneity.

a) The BDI-SF, 1instrument

Leticia et al. (2005) [36] validated the BDI-SF validated among 155 patients admitted to general medical wards in Brazil. The gold standard was based on the ICD-10 [28].

b) K-6, 1 instrument.

Tesfaye et al. (2009) validated the K-6 in 100 post natal women attending a general PHC clinic in Ethiopia. A psychiatric interview based on the DSM-IV [29] was used as the gold standard.

c) K-10, 4 instruments

The K-10 was validated at four PHC sites, one of which was an HIV PHC site. Fernandes et al. (2011) [45] validated the K-10 among 194 pregnant mothers at a rural prenatal clinic in India. Meanwhile Spies et al. (2009) [27] validated the K-10 in 429 HIV-infected adults in an HIV care centre in South Africa using the MINI as the gold standard. Baggaley et al. (2007) [11] validated a translated version of the K-10 in Burkina Faso among 61 women. A detailed diagnostic interview by a psychiatrist within 3 days of administering the K10 was the gold standard. Tesfaye et al. (2009) validated the K-10 in 100 post natal women attending a general PHC clinic in Ethiopia. A psychiatric interview based on the DSM-IV [29] was used as the gold standard.

d) PHQ-9, 1 instrument

The English language version of PHQ-9 was translated into Thai by Lotraku et al. (2008) [39], then back translated and adapted for use in Thailand. The PHQ-9 was then validated among 280 participants in a general PHC setting in Thailand.

e) EPDS, 5 instruments.

The EPDS was the most validated instrument in both pre and postnatal women. However, it should be noted that women accessing antenatal and postnatal care predominantly seek help for pregnancy related complaints, and may differ from persons attending general PHC. Despite such differences in the reason for seeking help at PHC, studies report a 10-20% prevalence of depression in postnatal women [49-51]. This high prevalence calls for the need to screen for depression in this population. We also report about these studies because such findings could be of interest to persons involved in women’s mental health research.

Fernandes et al. (2011) [45] validated the EPDS among 194 women in their third trimester of pregnancy at a rural prenatal clinic in Karnataka India. The gold standard against which the EPDS was validated was the ICD-10. In mainland China, Lau et al. (2010) [44] validated the Chinese version of the EPDS in 342 postnatal women, using the Structured Clinical Interview for DSM-III-R (SCID) [52] as gold standard.

In Zimbabwe, Africa, Chibanda et al. (2010) [43] validated the Shona version of EPDS scale among 210 postpartum HIV-infected and uninfected women attending two primary care clinics in peri-urban Harare, Zimbabwe. In Brazil, Figeuira et al. (2009) [37] validated the EPDS in a sub-sample of 245 mothers; the MINI was used as the gold standard.

Tesfaye et al. (2009) validated the EPDS in 100 post natal women attending a general PHC clinic in Ethiopia. A psychiatric interview based on the DSM-IV [29] was used as the gold standard.

f) Other brief (3) instruments

Puertas et al. (2004) [46] validated a visual analogue scale (VAS) and the GHQ-10 among 450 participants in India using the revised Clinical Interview Schedule (CIS-R) [53] as a gold standard. The CIS-R is based on the ICD-10 [28].

In Uganda, Muwhezi et al. (2007) [47] assessed the validity of a 4- item subjective well-being subscale (SWB) in detecting a major depressive illness. A total of 199 consecutive patients were enrolled at a PHC facility in Uganda, interviewed using the SWB and the MINI [48] as a gold standard.

**Table 1:** General description of the studies included in the systematic review

**Instrument**	**Author**	**Gold standard**	**Participant characteristics**	**Country of study**
BDI-SF ^**a**^	Leaticia et al. (2005)	ICD-10	Male/Female, in medical wards	Brazil
EPDS ^**b**^	Chibanda et al. (2010)	MINI	Female, postnatal HIV-PHC	Zimbabwe
EPDS ^**b**^	Lau et al. (2010)	SCID	Female, postnatal, general-PHC	China
EPDS ^**b**^	Fernandes et al. (2011)	MINI	Female, antenatal, general-PHC	India
EPDS ^**a**^	Figeuira et al. (2009)	MINI	Female, postnatal, general-PHC	Brazil
EPDS ^**b**^	Tesfaye et al. (2009)	DSM-IV	Female,postnatal, general-PHC	Ethiopia
CESD ^**a**^	Chisanga et al. (2011)	MINI	Male/Female HIV-PHC	Zambia
CESD ^**a**^	Myer et al. (2008)	MINI	Male/Female HIV-PHC	South Africa
K-6 ^**a**^	Tesfaye et al. (2009)	DSM-IV	Female,postnatal, general-PHC	Ethiopia
K-10 ^**c**^	Baggeley et al. (2007)	Psychiatrist	Female, post natal, general-PHC	Burkina
K-10 ^**b**^	Fernandes et al. (2011)	MINI	Female, antenatal, general-PHC	India
K-10 ^**b**^	Spies et al. (2009)	MINI	Male/Female HIV-PHC	South Africa
K-10 ^**a**^	Tesfaye et al. (2009)	DSM-IV	Female,postnatal, general-PHC	Ethiopia
PHQ-9 ^**a**^	Lotrakul et al. (2008)	SCID	Male/Female Family practice clinic.	Thailand
SRQ-20 ^**a**^	Stewart et al. (2009)	MINI	Female. Postnatal, General-PHC.	Malawi
VAS ^**b**^	Puertas et al. (2004)	CIS-R	Male/Female General-PHC	India
GHQ-10 ^**b**^	Puertas et al. (2004)	CIS-R	Male/Female General-PHC	India
SWB-4 ^**b**^	Muwhezi et al. (2007)	MINI	Male/Female General-PHC	Uganda
HSCL-25 ^**a**^	Kaaya et al. (2002)	SCID	Female, antenatal, HIV-PHC	Tanzania

*a denotes study of good quality, *b acceptable quality, and *c denotes study of fair quality.

**Table 2:** Parameters used to asses for heterogeneity of included studies

**Instrument**	**Author**	**No. of subjects**	**Prevalence**	**Sensitivity**	**Specificity**	**AUC**
BDI-SF	Leaticia et al. (2005)	155	20	100	83.1	0.98
EPDS	Chibanda et al. (2010)	210	30.4	88	87	0.82
EPDS	Lau et al. (2010)	342	22.2	81.2	80.7	0.89
EPDS	Fernandes et al. (2011)	194	14.4	100	84.9	0.95
EPDS	Figeuira et al. (2009)	245	26.9	86.4	91.1	0.94
EPDS	Tesfaye et al. (2009)	100	11.0	78.9	75.3	0.85
CESD	Chisanga et al. (2011)	659	13.1	73	76	0.78
CESD	Myer et al. (2008)	465	13.3	79	61	0.75
K-6	Tesfaye et al. (2009)	100	11.0	82.4	82.7	0.86
K-10	Baggeley et al. (2007)	61	43.3	74	76	0.77
K-10	Fernandes et al. (2011)	194	14.4	100	81.3	0.95
K-10	Spies et al. (2009)	425	53.3	67	77	0.77
K-10	Tesfaye et al. (2009)	100	11.2	84.2	77.8	0.87
PHQ-9	Lotrakul et al. (2008)	280	6.78	84	77	0.89
SRQ-20	Stewart et al. (2009)	114	30.5	59.2	85.4	0.85
VAS	Puertas et al. (2004)	450	48.5	75.5	63.3	0.69
GHQ-10	Puertas et al. (2004)	450	48.5	93.6	81.1	0.87
SWB-4	Muwhezi et al. (2007)	199	37.3	75.7	86.3	0.87
HSCL-25	Kaaya et al. (2002)	100	11.1	89	80	0.86

### Longer scales

a) CES-D, 2 instruments

In Zambia, Africa, Chisanga et al. (2011) [38] conducted a cross-sectional study in 16 primary level care clinics and validated the CES-D in PLWHA who had tuberculosis and were starting ART. Chisanga validated the CES-D against the MINI [48] as gold standard.

Myer et al. (2008) [26] validated the CES-D among 465 participants individuals had enrolled into HIV care in South Africa. He used the MINI as gold standard.

b) SRQ-20, 1 instrument

In Malawi, Stewart et al. (2009) [40] validated the Chichewa version of the Self Reporting Questionnaire (SRQ) was validated among 114 subjects at a PHC site. This instrument went through a process of forward and back translation.

c) Other long instruments

Kaaya et al. (2002) [41] validated the Hopkins Symptom Checklist-25 (HSCL-25) among 99 women who were pregnant and HIV positive in Tanzania. The gold standard was the SCID [52].

### Analysis for the presence of heterogeneity between studies

We used the ‘meta’ commands of STATA to generate the forest plots and assess for heterogeneity. The test for heterogeneity using a random effects analysis model yielded a statistically significant result. Heterogeneity chi-squared = 189.23, p = 0.000 on 18 degrees of freedom.

Statistically significant heterogeneity meant we could not continue with the meta-analysis and report the results as pooled estimates.

## Discussion

We present the first systematic review comparing the accuracies of brief and long depression screening instruments which have been validated in LMIC settings. In this review, we found evidence to show that within LMIC, a number of depressed patients are identified using screening instruments at PHC settings. The prevalence figures reported in the included studies also vary widely across PHC settings within LMIC.

We found statistically significant heterogeneity between studies and could not conduct a meta-analysis to the end. The heterogeneity across studies could be the result of methodological differences in validation of instruments. For example, we found that a single instrument could be validated using different reference standards, producing different cut off scores and AUC scores. The CESD and EPDS were such examples in our review [26,38,43,45]. In addition, these studies were conducted across continents and settings with different cultures, languages and resources.

Both brief and longer scales showed moderate to high accuracy, with AUC ranging from 0.69-0.99. Our review found evidence to show that brief scales including the PHQ-9, BDI-SF, K-6, K-10, EPDS, and GHQ-12 were as accurate as the longer ones like the CES-D, HSCL, and BDI. These findings are in agreement with previous reviews which assessed the accuracy of depression screening instruments in HIC [6,14]. For example, a review of instruments validated in the Spanish language reported overall sensitivity and specificity in the range of 70-90% [13]. Studies with AUC’s values of 0.50 to 0.70 are generally considered of low accuracy, 0.70 to 0.90 as having moderate accuracy, and those with AUC ≥ 0.90 as highly accurate [54,55]. Of the instruments studied, the EPDS shows acceptable accuracy in detecting depression among pre and post-natal women, which was in agreement with a previous systematic review [50]. Among HIV clinic populations, the HSCL-25 [41] showed the highest sensitivity at 89%.

No single instrument was superior to another in our review, perhaps due the relatively small number of studies with any particular instrument. Previous reviews that have assessed diagnostic accuracy of depression instruments were equally unable to recommend a single instrument for use in PHC [15,50].

### Limitations

A number of limitations should be acknowledged. For example, we did not include studies that were not published in English. That said, our literature review did not return any studies in other languages that appeared to meet our inclusion criteria. While some studies published in non-indexed journals may have escaped notice, there has been an increase in indexed journals in LMIC in recent years, and most studies of quality should therefore have been captured.

Secondly, we didn’t include in our review instruments which had been used to screen for the whole range of psychiatric morbidity, limiting our scope to those that had been validated for depression only. The inclusion of such scales which had screened for both depression and anxiety disorders could have been more informative; however, such criteria could have turned up numerous studies which may have been difficult to synthesize. Much as the K-10, GHQ and SRQ-20 instruments asses for common mental disorders including anxiety, depression and psychological distress, we only included them if they had been used to screen for depression.

## Conclusion

Brief instruments are as accurate as the longer ones in detecting depression in both general and HIV-PHC settings. The brief nature of a screening instrument (BDI-SF, PHQ-10, and K-10) gives it the edge over longer scales like the CES-D due the short duration in which it can be administered. However, the fact that ultra-brief scales such the K-6 and BDI-SF don’t encompass a whole range of depressive symptoms including suicide, the use of such scales needs to be followed up with detailed psychiatric diagnostic interviews. The K-6 was shown to be as accurate as the K-10 in the study by Tesfaye et al. (2009).

Other scales such as the EPDS may be the instrument of choice in particular populations (e.g. postnatal mothers).

## Competing interest

The authors declare no competing interest, financial or otherwise.

## Authors’ contributions

DA, EO and TA independently abstracted all papers. DA read all the papers. JJ scrutinized all included and excluded studies. In the event of ambiguity, DJS was the arbitrator. DJS and SM were regularly consulted in the conceptualization of the paper. All authors read and approved the final manuscript.

## Pre-publication history

The pre-publication history for this paper can be accessed here:

http://www.biomedcentral.com/1471-244X/12/187/prepub
